# Vestibular function in superficial siderosis

**DOI:** 10.1186/1472-6815-13-5

**Published:** 2013-04-23

**Authors:** Toru Miwa, Ryosei Minoda, Hidetake Matsuyoshi

**Affiliations:** 1 Department of Otolaryngology and Head and Neck Surgery, Kumamoto University, 1-1-1 Honjo, Kumamoto, Japan; 2 Matsubase ENT Clinic, Kumamoto, Japan

**Keywords:** Hemosiderin, Superficial siderosis, Vertigo, Vestibular function, Clinical neurology examination

## Abstract

**Background:**

Superficial siderosis (SS) is caused by repeated or continuous bleeding into the subarachnoid space that results in iron from hemoglobin (hemosiderin) being deposited on the surface of the brain. Clinically, the condition is characterized by sensorineural deafness, ataxia, and pyramidal signs. However the mechanism of peripheral vestibular disturbance was not revealed. We show the vestibular function of SS patients, and shed light on saccule-inferior vestibular nerve.

**Methods:**

Over the past 9 years, 5 patients were definitively diagnosed with SS by MRI in our department. These patients were subjected to balance testing.

**Results:**

Vestibular evoked myogenic potential (VEMP) was observed in patients who had suffered from SS for a short period but tended to be diminished or absent in patients who had suffered from the condition for a longer period.

**Conclusions:**

These findings in SS patients suggest that saccule-inferior vestibular function is maintained at early stages of the disorder. Our study may help to clarify the mechanism of SS.

## Background

Superficial siderosis (SS) of the central nervous system (CNS) is caused by repeated or continued bleeding into the subarachnoid space resulting in the deposition of iron from hemosiderin onto the surface of the brain [1,2]. SS is classified into two groups based on its cause: *idiopathic,* for which the source of the bleeding is not identified, and *symptomatic,* for which a source of bleeding is identified. A review of 270 SS patients identified *idiopathic SS* in 35% of cases, while the causes of *symptomatic SS* included current or previous CNS tumors (21%), head or back trauma (13%), arteriovenous malformations/aneurysms (9%), post-surgical changes related to neurosurgeries (7%), brachial plexus injury (6%), amyloid angiopathy (AA) (3%), and other chronic subdural hematomas (6%) [3]. Clinically, SS is characterized by sensorineural deafness, ataxia, pyramidal signs, and dementia.

Vestibular deficits due to SS have rarely been reported in the otolaryngological literature because early reports noted the selective deposition of hemosiderin around the CNS and/or the 8th nerve in contact with the cerebrospinal fluid, most notably the cerebellum, brainstem, lining of the ventricles, and spinal cord [1,4]. These deposits around CNS structures and/or the 8th nerve were considered to be the changes most responsible for the disequilibrium of SS. However, *Fukiyama et al.* reported that the cause of impaired balance lies in the damage to the inner hair cells. The deposition of hemosiderin in the inner ear and the subsequent fibrosis thicken the dura mater, decreasing peripheral blood flow in the inner ear [5].

Over the past 9 years, we have performed balance testing on 483 patients who suffer from impaired balance or hearing loss. Among them, 5 patients were definitively diagnosed with SS by magnetic resonance imaging (MRI). This study provides a retrospective report on these five patients. We examined the root cause of the vestibular deficits due to SS through otolaryngological vestibular balance testing.

Vestibular evoked myogenic potential (VEMP) testing is frequently utilized in the assessment of a variety of vestibular etiologies. The VEMP response is obtained by measuring the release of the sternocleidomastoid (SCM) muscle from a contracted state provoked by delivering auditory stimuli to the ipsilateral ear [6]. VEMP responses are considered to be a reflection of vestibulospinal projections to the neck, which offer information regarding the saccule and inferior vestibular nerve integrity [7,8]. The caloric test, on the other hand, is considered to be a reflection of the otolith and lateral semicircular canal functions. This test provides information regarding the utricle and superior vestibular nerve [9]. Few reports have discussed VEMP in SS patients because VEMP is a relatively new test and SS is a rare disease. We performed VEMP testing on all patients to assess their saccule-inferior vestibular function.

## Methods

Over the past 9 years, we have performed balance testing on 483 patients suffering from impaired balance or hearing loss. Among them, five patients were diagnosed with SS by MRI. The patient ages ranged from 53 to 79 years (mean: 64.5 ± 12.6 years), and the patients included three males and two females. SS is classified into two groups based on its causes, *idiopathic* and *symptomatic*.

Balance testing consisted of dynamic balance testing via walking and stepping tests and static balance testing via Mann’s test and stabilometry (eyes open and eyes closed). Testing for gaze nystagmus, spontaneous nystagmus, and positional and positioning nystagmus was performed using an infrared camera. Electronystagmography (ENG) was utilized for an eye-tracking test (ETT), an optokinetic nystagmus (OKN) test, and a caloric test. During the caloric test, stimulation was provided by irrigation with 5 ml cold water (20 degrees Celsius) for 20 sec. The maximum slow-phase velocity was measured based on ENG recordings. In addition, a VEMP test was performed. The VEMP test featured 105-dB nHL clicks, 0.1 ms in duration, with a stimulation frequency of 5 Hz and an analysis time of 50 ms; the responses to 200 stimuli were averaged, and a band-pass filter of 20–2000 Hz was used. The patient’s neck was rotated during the testing. The hearing tests consisted of pure-tone audiometry, speech audiometry, and a distortion product otoacoustic emission (DPOAE) test.

The MRI of the brain was performed on 3.0 T clinical units equipped with head coils. T2-weighted and T2*-weighted imaging were performed in the axial planes.

### Cases

#### I. Idiopathic SS

**Figure 1:**
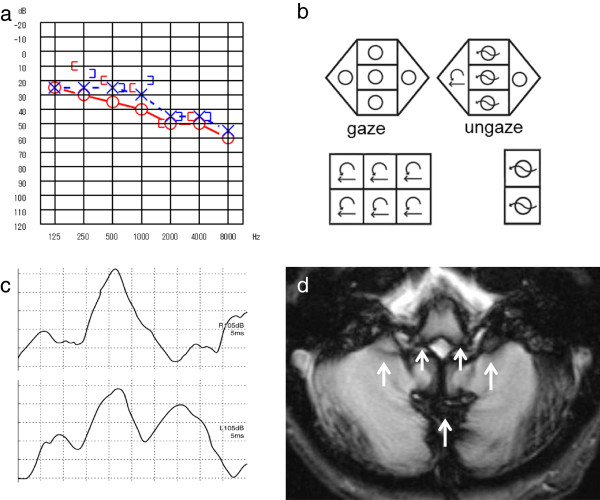
**Patient 1. a**. Pure-tone audiometry: bilateral moderate sensorineural hearing loss. **b**. Nystagmus test: mixed horizontal and rotatory nystagmus to the right during the supine roll test. **c**. VEMP: bilateral normal. **d**. MRI: T2*-weighted images revealed hemosiderosis around the brainstem and the cerebellum, partially the lateral Sylvian fissure and longitudinal cerebral fissure and the base of the brain, and the 8th nerve (arrow).

Case 1: A 53-age-year old woman, without medical history, presented with progressive bilateral hearing loss with tinnitus, headaches, and dizziness for several months. The otoneurologic examination showed bilateral moderate sensorineural hearing loss (Figure 1a). Body swaying with her eyes open was revealed by both the dynamic and static postural tests. The ENG showed that OKN was poor on the left, with mixed horizontal and rotatory nystagmus to the right during the supine roll test (Figure 1b), and right hyporeflexia was observed in the caloric test. No findings were revealed by the VEMP responses (Figure 1c). Magnetic resonance imaging (MRI) of the brain was performed in the axial planes. The MRI axial T2*-weighted images showed hemosiderosis around the brainstem and the cerebellum, partially around the surface of the lateral Sylvian fissure and the longitudinal cerebral fissure and the base of the brain, and the 8th nerve (Figure 1d).

Cause of impaired balance: CNS damage + utricle-superior vestibular dysfunction.

**Figure 2:**
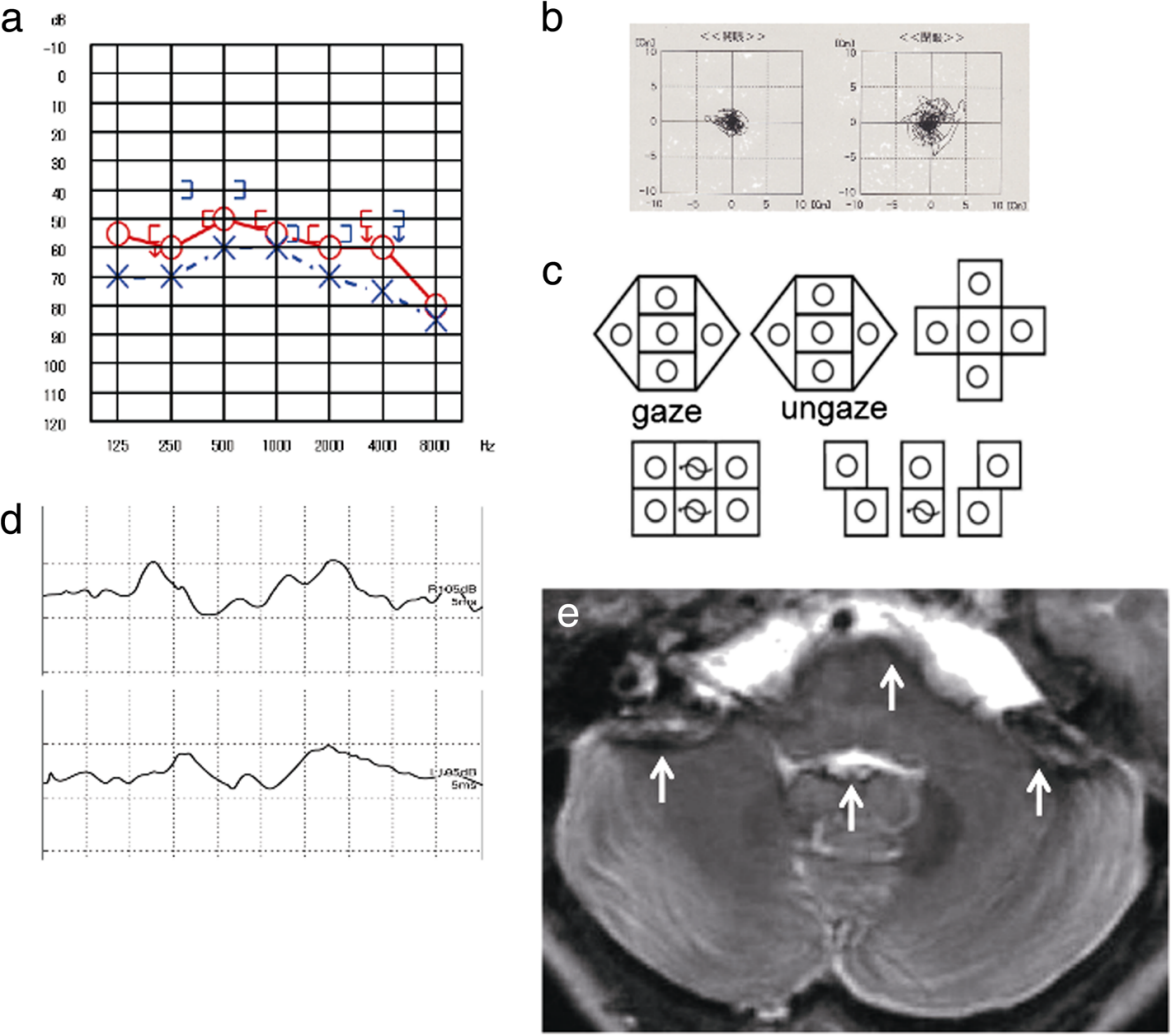
**Patient 2. a**. Pure-tone audiometry: moderate sensorineural hearing loss on the right and moderate mixed conductive-sensorineural hearing loss on the left. **b**. Stabilometry: body swaying with her eyes open. **c**. Nystagmus test: horizontal nystagmus to the right during the supine roll test. **d**. VEMP: bilateral absent. **e**. MRI: T2*-weighted images revealed hemosiderosis around the brainstem and the cerebellum, partially the lateral Sylvian fissure and longitudinal cerebral fissure (arrow).

Case 2: A 71-age-year old woman, without medical history, presented with progressive bilateral hearing loss and dizziness for a few years. The otoneurologic examination showed bilateral moderate sensorineural hearing loss (Figure 2a). Speech perception was worse on the left. Body swaying with her eyes open was revealed by both dynamic and static postural tests (Figure 2b). The ENG revealed abnormal eye movements with saccadic ocular pursuit, pathological OKN, and horizontal nystagmus to the right during the supine roll test (Figure 2c), and bilateral areflexia was observed in the caloric test. Bilateral VEMP responses were absent (Figure 2d). MRI T2*-weighted images showed hemosiderosis around the brainstem and the cerebellum, as well as partially the lateral Sylvian fissure and the longitudinal cerebral fissure (Figure 2e).

Cause of impaired balance: CNS damage + utricle-superior and saccule-inferior vestibular dysfunction.

#### II. Symptomatic SS

**Figure 3:**
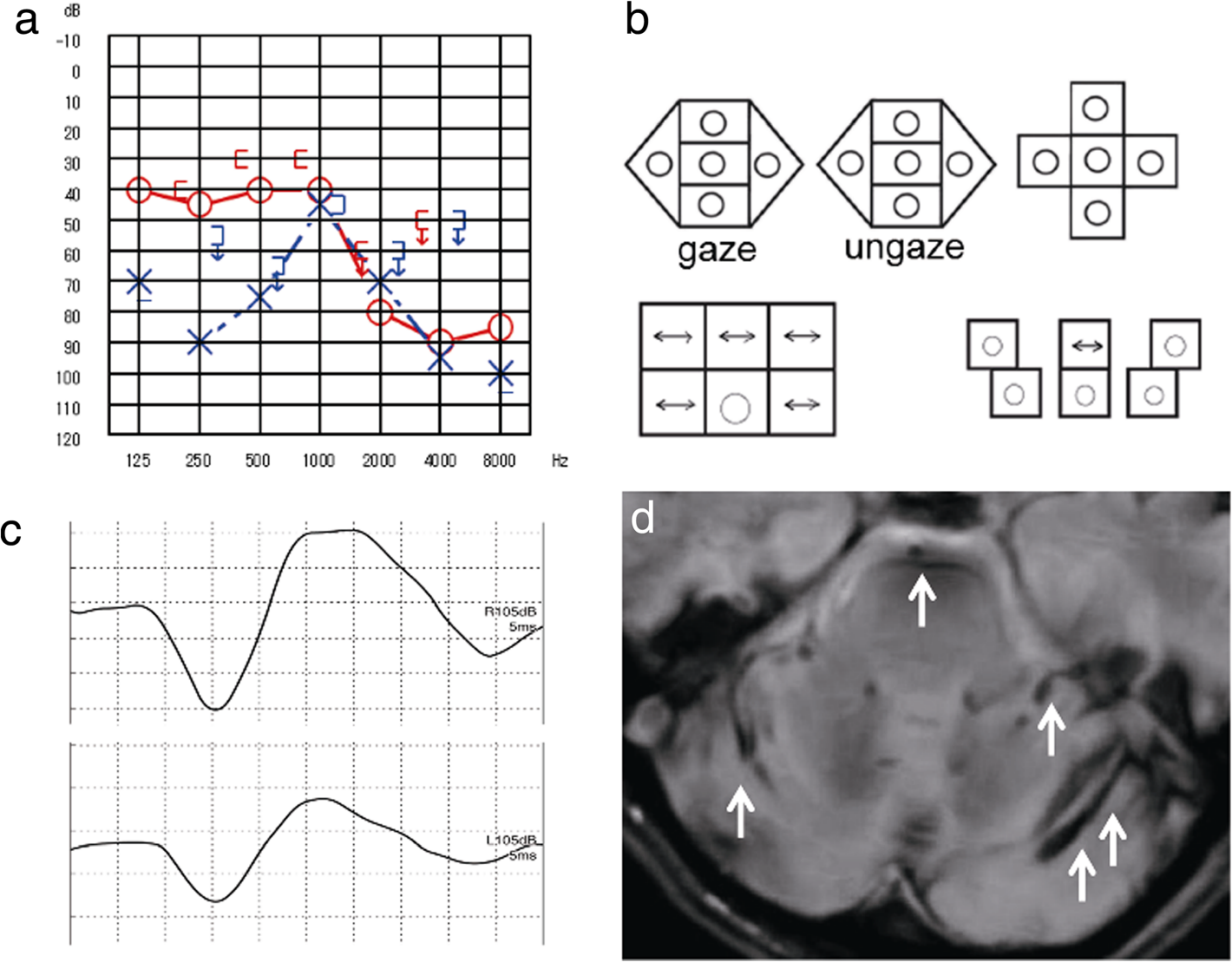
**Patient 3. a**. Pure-tone audiometry: mild sloping sensorineural hearing loss on the right. Reverse-cookie-bite sensorineural hearing loss at 1000 Hz on the left. **b**. Nystagmus test: pendular nystagmus during the supine roll test. **c**. VEMP: bilateral normal. **d**. MRI: T2-weighted images revealed hemosiderosis around the cerebellum, the medulla oblongata, and the right temporal lobe (arrow).

Case 3: A 55-age-year old man underwent brain surgery to remove a cavernous hemangioma of the right ventricle due to bleeding. Several days later, he complained of progressive bilateral hearing loss and dizziness. The otoneurologic examination showed bilateral moderate to severe sensorineural hearing loss (Figure 3a). Speech perception was worse on the left. Bilateral DPOAE responses were diminished. Body swaying with his eyes closed was revealed on the dynamic postural test. The ENG revealed abnormal eye movements with saccadic ocular pursuit, pathological OKN, pendular nystagmus during the supine roll test (Figure 3b), right hyporeflexia in the caloric test and the disappearance of visual suppression. No findings were revealed by the VEMP responses (Figure 3c). The MRI axial T2-weighted images showed hemosiderosis around the cerebellum, the medulla oblongata, and the right temporal lobe (Figure 3d).

Cause of impaired balance: CNS damage + utricle-superior vestibular dysfunction.

**Figure 4:**
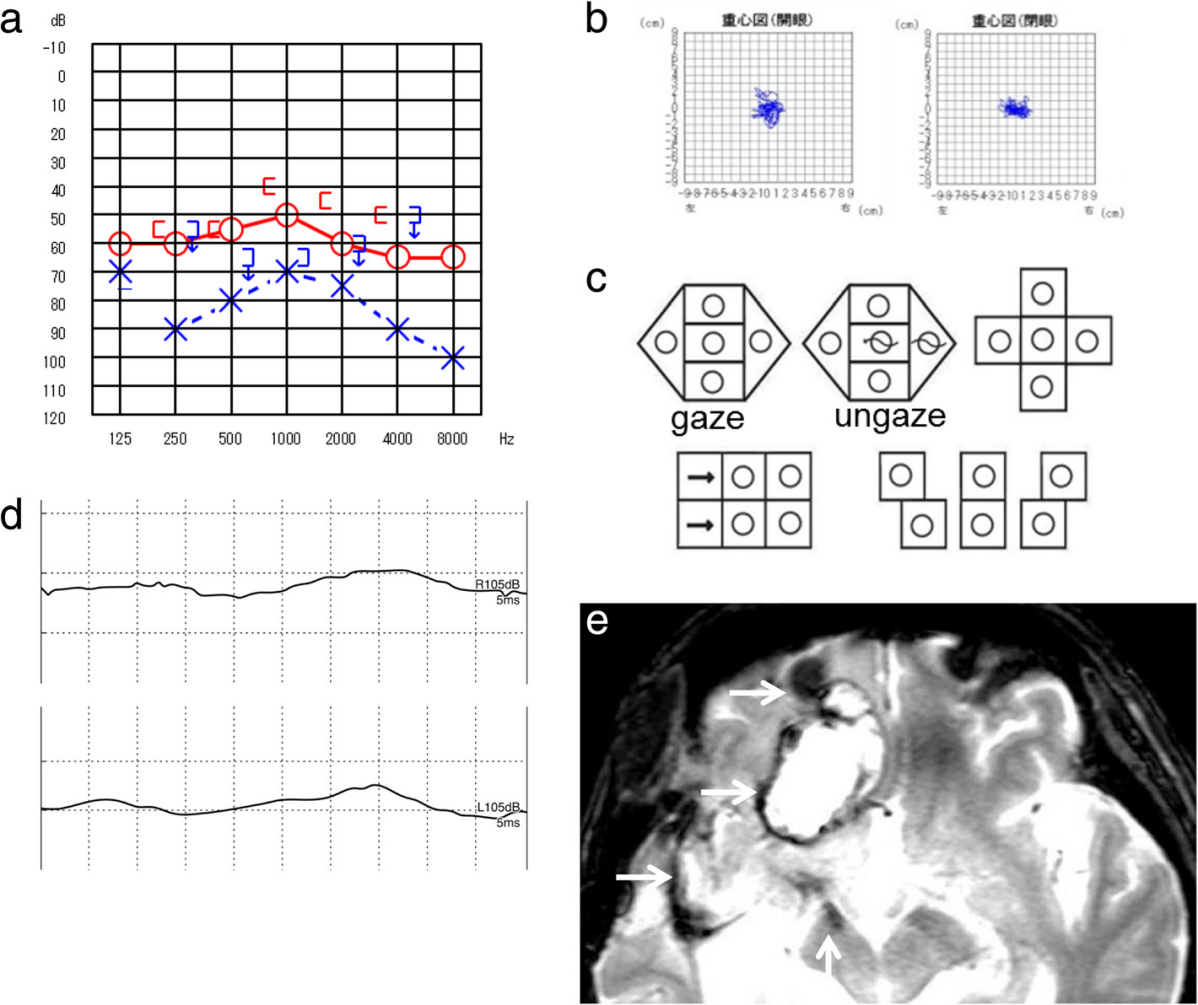
**Patient 4. a**. Pure-tone audiometry: moderate sensorineural hearing loss on the right and severe sensorineural hearing loss on the left. **b**. Stabilometry: body swaying with his eyes open. **c**. Nystagmus test: horizontal nystagmus to the left during the supine roll test. **d**. VEMP: bilateral absent. **e**. MRI: T2*-weighted images revealed hemosiderosis around the right temporal lobe and the basal ganglia (arrow).

Case 4: A 73-age-year old man presented with progressive hearing loss with tinnitus on the left accompanied by vertigo for a few years. His medical history revealed that brain surgery was performed for a cyst in the right temporal lobe when he was 50 years old. The otoneurologic examination showed moderate sensorineural hearing loss on the right and severe sensorineural hearing loss on the left (Figure 4a). Bilateral speech perception was diminished. Bilateral DPOAE responses were absent. Body swaying with his eyes open was revealed by the static postural test (Figure 4b). The ENG revealed abnormal eye movements with saccadic ocular pursuit, pathological OKN, horizontal nystagmus to the left during the supine roll test (Figure 2c), and bilateral hyporeflexia on the caloric test. Bilateral VEMP responses were absent (Figure 2d). The MRI T2*-weighted images showed hemosiderosis around the right temporal lobe and the basal ganglia (Figure 4e).

Cause of impaired balance: CNS damage + utricle-superior and saccule-inferior vestibular dysfunction.

**Figure 5:**
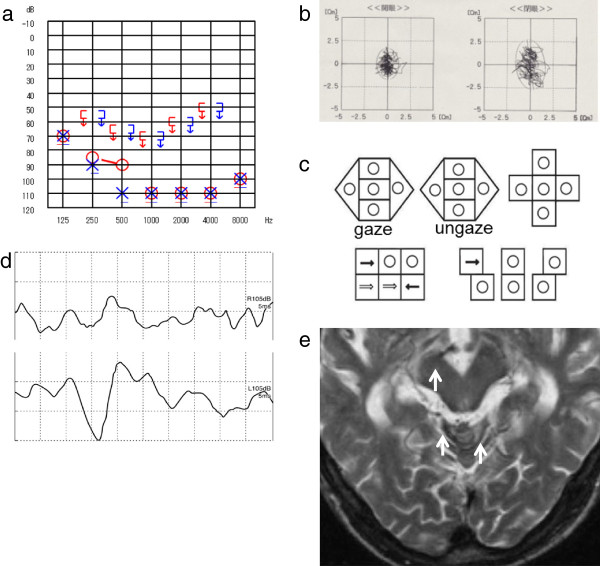
**Patient 5. a**. Pure-tone audiometry: bilateral deafness. **b**. Stabilometry: normal. **c**. Nystagmus test: vertical nystagmus upward during the supine roll test. **d**. VEMP: diminished on the left. **e**. MRI: T2-weighted images revealed hemosiderosis around the brainstem and the cerebellum (arrow).

Case 5: A 79-age-year old man presented with dizziness and progressive bilateral hearing loss beginning when he was 62 years old. His medical history revealed that heart surgery was performed for a mitral valve dysfunction at 57 years old, and he suffered from hypertension. The patient was on anti-coagulation medication and a Ca^2+^-blocker. The otoneurologic examination showed bilateral deafness (Figure 5a). Bilateral DPOAE responses were absent. Body swaying was normal on the static postural test (Figure 5b), but not on the dynamic postural test. The ENG revealed abnormal eye movements with saccadic ocular pursuit, pathological OKN, vertical nystagmus upward during the supine roll test (Figure 5c), and bilateral areflexia on the caloric test. The VEMP responses were diminished on the left (Figure 2d). The MRI T2-weighted images showed hemosiderosis around the brainstem and cerebellum (Figure 5e).

Cause of impaired balance: CNS damage + utricle-superior and saccule-inferior vestibular dysfunction.

Summary of five patients were indicated in Table 1.

**Table 1:** Summary of five patients

**Pt**	**Age Sex**	**Chief complaint**	**Duration of disease**	**Cause of bleeding**	**Audiologic data**			**Vestibular data**							**MRI Deposit of hemosiderin**		**Cause of impaired balance**
					**PTA (dB)**	**Speech**	**DPOAE**	**Dynamic Posturo- graphy**	**Romberg sign**	**Nystagmus**	**ETT**	**OKN**	**Caloric**	**VEMP**	**CNS**	**8th**	
1	53 F	Bil HL Dizziness Tinnitus Headache	Several months	Unknown	Rt41.7 Lt33.3	-	-	AN	+	mixed horizontal androtatory nystagmus to the right	N	AN	Rt: Hypo-reflexia Lt: N	N	+	+	CNS
2	71 F	Bil HL, Dizziness	2-3 years	Unknown	Rt55.0 Lt63.3	-	-	AN	+	horizontal nystagmus to the right	N	AN	Areflexia	Absent	+	-	CNS +SV +IV
3	55M	Bil HL	Several weeks	Post brain surgery	Rt53.3 Lt63.3	Rt 70% (70dB) Lt10% (100dB)	none	N	+	pendular nystagmus	AN	AN	Rt: Hypo- reflexia Lt: N	N	+	-	CNS +SV
4	73M	Lt HL Vertigo Tinnitus	2-3 years	Post brain surgery	Rt55.0 Lt75.0	Rt 65% (90dB) Lt 70% (100dB)	Absent	N	+	horizontal nystagmus to the left	AN	AN	Areflexia	Absent	-	-	CNS +SV +IV
5	79M	Bil HL	17 years	HT	Deaf	-	none	AN	-	Vertical nystagmus upward	AN	AN	Areflexia	Lt Absent	+	-	CNS +SV +IV

Saccule-inferior vestibular function is maintained at early stages of the superficial siderosis patients.

Abbreviation: HL: Hearing loss, HT: Hypertension, PTA: Pure Tone Audiometry, DPOAE: Distortion product otoacoustic emissions, N: normal, AN: abnormal,

ETT: Eye tracking test, OPN: Optokinetic nystagmus, VEMP: Vestibular evoked myogenic potential, CNS: Central nervous system,

SV: Superior vestibular function, IV: Inferior vestibular function.

## Discussion

SS is caused by repeated or continued bleeding into the subarachnoid space that results in the deposition of iron from hemosiderin onto the surface of the brain [1,2]. Clinically, this condition is characterized by sensorineural deafness, ataxia, pyramidal signs, and dementia. In our study, all patients presented sensorineural hearing loss and impaired balance, but none suffered from dementia. The first report of SS was provided by Hamill in 1908 [1], and the autopsies of two affected patients were reported in 1940 [4]. The histopathological findings of these autopsies showed hemosiderin deposition at the surface of the CNS in close proximity to the cerebrospinal fluid spaces. The deposition of hemosiderin is associated with gliosis, neuronal loss, and demyelination [10]. The widespread use of MRI has enabled physicians to diagnose SS without a biopsy and to discover *symptomatic* cases. Descriptions of a total of 270 cases of SS have been published [3]. However, vestibular deficits due to SS have been rarely reported in the otolaryngological literature because early reports noted the selective deposition of hemosiderin around the CNS in contact with the cerebrospinal fluid, most notably around the cerebellum, brainstem, lining of the ventricles, and spinal cord [1,4]. These CNS structures were considered to be the most affected by the disequilibrium of SS. In our study, patients 3 and 4 presented brain tumors with bleeding. They suffered from sensorineural hearing loss and disequilibrium, in the form of ataxia with their eyes open and dizziness or vertigo with their eyes closed. We concluded that the hemosiderosis around the CNS caused their impaired balance.

Recently, vestibular deficits due to SS have been reported due to hemosiderin deposition around the 8th cranial nerve and cochlear damage [5,10-12]. The 8th nerve is described as particularly vulnerable. Hemosiderin formation occurs mainly within the microglia as they synthesize ferritin, so hemosiderin is taken up selectively by CNS areas rich in microglia and areas close to the CSF flow [13]. The 8th nerve is rich in microglia, and it remains within the CNS until it enters the internal acoustic canal, a relatively long distance outside of the brain, making it vulnerable to the damaging effects of chronic subarachnoid hemorrhage within the CNS [14,15]. The optic nerves (2nd) remain wholly within the CNS, but they are somehow spared from the damaging effects of heme, possibly because of the absence of heme-absorbing glia along their tracts. In addition, cochlear damage can cause the vestibular deficits of SS. Specifically, temporal bone histopathology has revealed the atrophy of the superior and inferior vestibular nerves and the loss of hair cells [16], and caloric tests have revealed hyporeflexia [17-23]. *Fukiyama et al.* reported that one cause of impaired balance is damage to the inner hair cells by the deposition of hemosiderin in the inner ear. Subsequent fibrosis thickens the dura mater, and the peripheral blood flow in the inner ear is decreased [5]. Thus, central nervous damage, 8th cranial nerve damage, and cochlear damage are considered to be the causes of impaired balance due to SS [5,11,12,16]. In our study, patients 1, 2, and 5 suffered from disequilibrium characterized by cerebellar ataxia with their eyes open and dizziness with their eyes closed. Further vestibular examination proved that they had central nervous damage, 8th cranial nerve damage, and/or cochlea damage.

Vestibular deficits due to SS are rarely reported in the otolaryngological literature. To date, 16 cases have been reported for vestibular assessment [3,24]. Among them, VEMP findings were reported in only 1 patient [23]. We performed balance testing on five patients suffering from SS. All of the patients were found to have both central nervous damage and peripheral vestibular damage; this finding agrees with other reports. In addition, we performed the VEMP test on all patients to assess their saccule-inferior vestibular function. The results showed that VEMP responses were normal for patients who had suffered from SS for a short period but tended to be diminished or absent in patients who had suffered from SS for a longer period. This result indicated that saccule-inferior vestibular function was maintained in patients early in SS. In a previously reported case, the patient had suffered from SS for a period of 21 years; the caloric test in this report revealed bilateral hyporeflexia, and the absence of bilateral VEMP responses [23]. Subarachnoid hemorrhaging must persist for at least several months to overwhelm the body’s clearance mechanisms and cause symptoms. Depending on the volume and location of the bleeding, this process can take considerably longer, so our VEMP results are consistent with the hemosiderosis mechanism. Similarly, utricle-superior vestibular function was diminished or absent in all of the patients in our study. Vestibular damage to the utricle-superior vestibular system tended to precede vestibular damage to the saccule-inferior vestibular system. Anatomically, the superior vestibular nerve is longer and travels through smaller osseous neural canals [25]. Thus, more surface area of the nerve is in contact with cerebrospinal fluid, so hemosiderin is more readily deposited, and constriction and impaired blood flow are more likely to develop. Thus, the superior vestibular nerve is more susceptible to damage than the inferior vestibular nerve. However, in our study, the MRI findings revealed hemosiderosis of the 8th nerve in only 1 patient (patient 1). This finding implies that the causes of the balance impairments are cochlear damage, likely caused by constriction and impaired blood flow, rather than vestibular nerve damage due to the deposition of hemosiderin. This result corroborates the report by *Fukiyama et al.* indicating that cochlear damage due to SS is caused by impaired blood flow in the inner ear [5].

The treatment of SS involves identifying the cause of the bleeding and treatment of the underlying cause when it is apparent. When the cause is not apparent, a chelating agent is administered to deplete iron, or a hemostatic agent is administered to stop bleeding. However, such treatment is inadequate, and effective therapies have yet to be established [17,26]. Approximately 40% of cases of bleeding due to SS are *idiopathic*[17]. Other causes of bleeding, reported as *symptomatic*, include current or previous CNS tumors, head or back trauma, arteriovenous malformations/aneurysms, post-surgical changes related to neurosurgeries, brachial plexus injury, amyloid angiopathy (AA), and other chronic subdural hematomas [26-30]. In patients with AA and/or hypertension, microbleeds into the subarachnoid space continue due to the fragility of the vessels in the meninges [31,32]. In our study, the cause of bleeding was apparent in patients 2 and 4, but they had been previously treated when initially examined for this study. We considered that the cause of the bleeding in patient 5 could have been microbleeds due to chronic hypertension because he presented no signs of AA and was medicated with an anti-coagulation drug. In the other two patients, the causes of the bleeding were not apparent.

The prognosis for SS is relatively good, with some patients surviving 20 to 30 years after developing the condition [17,28,33]. Sensorineural deafness often progresses, and quality of life (QOL) is markedly diminished. Recently, many SS patients have received cochlear implants [18,19,34-40]. Because the impaired balance is due to central nervous damage and peripheral vestibular damage, acquiring or achieving balance via the vestibuloocular reflex often proves difficult. There is no compensatory mechanism, and symptoms will persist, often diminishing QOL. The course of the current patients could not be followed, so the diminishing of vestibular function could not be ascertained. However, periodic balance testing could likely facilitate the observation of the diminishing of vestibular function.

This report revealed that the lesions responsible for impaired balance due to SS lie in the central nervous system and the peripheral vestibular system. Vestibular function, particularly the saccule-inferior vestibular function, diminishes the longer a patient suffers from the condition. Nevertheless, the pathology of SS remains rather unclear, and effective therapies have yet to be established. These issues must be examined in the future by assembling and analyzing additional cases.

## Informed consent

Written informed consent documents were obtained from the five patients for publication of this case report and any accompanying images. The copies of the written consent documents are available for review by the Editor-in-Chief of this journal.

## Competing interests

The authors declare that they have no competing interests.

## Authors’ contributions

TM and HM conceived, designed, and carried out the experiments TM analyzed the data and wrote the manuscript. RM conceived of the study, and participated in its coordination. All authors read and approved the final manuscript.

## Pre-publication history

The pre-publication history for this paper can be accessed here:

http://www.biomedcentral.com/1472-6815/13/5/prepub
